# Efficacy of the transfluthrin-based personal insect repellent kit (PIRK) against the ixodid ticks *Ixodes scapularis, Amblyomma americanum* and *Dermacentor variabilis*

**DOI:** 10.1016/j.crpvbd.2021.100070

**Published:** 2021-12-20

**Authors:** Maria V. Murgia, Jasleen Kaur, Laurie Widder, Catherine A. Hill

**Affiliations:** a Purdue University, Department of Entomology, West Lafayette, IN 47907-2089, USA; b Widder Brothers, Inc., New York, NY 10019, USA; c Purdue Institute for Inflammation, Immunology and Infectious Disease, West Lafayette, IN 47907-2089, USA

**Keywords:** Tick, Personal insect repellent kit (PIRK), Transfluthrin, *Ixodes*, *Amblyomma*, *Dermacentor*

## Abstract

An assay series was performed to assess the contact and spatial efficacy of the Personal Insect Repellent Kit (PIRK) against three species of ixodid ticks. The PIRK, a portable, passive device comprised of an inert physical substrate incorporated with the active ingredient (AI) transfluthrin (TF), has demonstrated spatial efficacy against flying insects, including three species of mosquitoes, sand flies and stable flies. The device is the only TF end-use product registered with the EPA. Here we report the first studies to explore potential of the PIRK to control *Ixodes scapularis*, *Amblyomma americanum* and *Dermacentor variabilis*. Dose-response assays confirmed toxicity of TF to larvae of all species in the μg/ml range following a 30-min exposure period. Nymphs and adults exhibited irritancy and avoidance behaviors on contact with the PIRK. Greater than 90% knockdown (KD) of *I. scapularis* nymphs and adults was observed after a 10-s exposure, and of *A. americanum* nymphs and adults after 10-s and 120-s exposure, respectively. Additionally, greater than 90% mortality was observed in *I. scapularis* nymphs and adults after 10-s and 40-s exposure, respectively. In spatial assays, the PIRK caused KD and post-exposure mortality of adult female *I. scapularis* exposed at a range of 5–28 cm. These results suggest both contact and spatial capacity of the PIRK, with greatest potency to nymphs *versus* adults and the prostriate tick *I. scapularis versus* the metastriate species *A. americanum* and *D. variabilis.* Future studies will explore spatial activity at a range of distances and exposure times, in the presence and absence of host cues and under semi-field conditions.

## Highlights

• First study to evaluate efficacy of the PIRK to species of *Ixodes*, *Amblyomma* and *Dermacentor.*
• Contact with the PIRK caused irritation, knockdown and mortality of adult ticks.
• Knock-down and contact toxicity following PIRK exposure was species-dependent.
• *Ixodes scapularis* exhibited greatest susceptibility to the PIRK and transfluthrin.
• Spatial activity observed in *I. scapularis* adults exposed to the PIRK at short ranges.

## Graphical abstract

**Figure:**
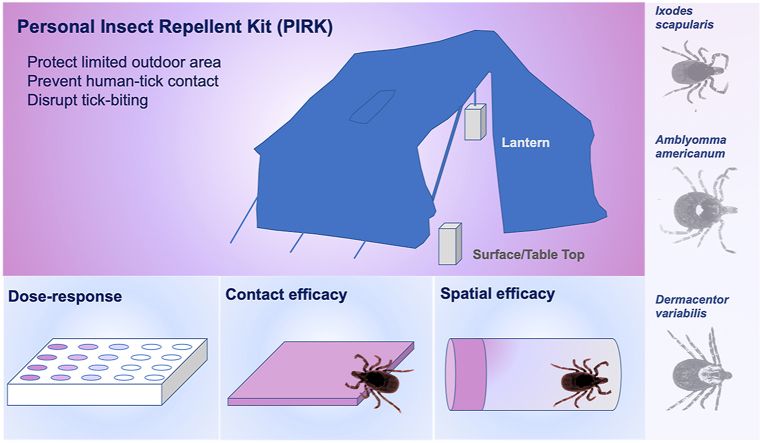

## 1 Introduction

Tick-borne diseases (TBDs) represent threats to public health, national defense and biosecurity. Species of hard ticks (family Ixodidae) transmit bacteria, viruses and protozoans that cause disease in humans and animals. The tick-borne Crimean-Congo hemorrhagic fever virus is considered an operational threat to the USA military abroad. In the USA, TBD cases more than doubled and seven new TBDs were recognized in the period 2004–2016 (Rosenberg et al., 2018). The increase in incidence of new and re-emerging TBDs is a global phenomenon, attributed to expansions in tick populations, among other factors (Medlock et al., 2013). In addition, there is growing concern regarding the risks posed by invasive tick species such as the Asian long-horned tick, *Haemaphysalis longicornis.* First detected in the USA in 2017 and considered established in multiple states (Rainey et al., 2018; Hutcheson et al., 2019), this species can build to high numbers *via* parthenogenesis and is the recognized vector of human and animal pathogens.

Control of TBDs is complicated by lack of diagnostics, therapeutics, and protective vaccines. Prevention of infectious bites relies on personal protection *via* EPA-approved topical repellents such as DEET, permethrin-treated clothing, and regular tick checks following outdoor activities (CDC, 2020; Eisen, 2021). Efficacy studies of permethrin-treated textiles have shown contact irritancy, inhibition of host-seeking behavior and post-exposure mortality of the black legged tick, *I. scapularis*, the American dog tick, *Dermacentor variabilis*, and the lone star tick, *Amblyomma americanum* (Eisen et al., 2017; Prose et al., 2018). While these approaches can reduce bite risk, they do not offer complete protection, and compliance is typically low (Bibbs et al., 2018; Connally et al., 2019). Innovative tick-bite prevention technologies that are safe for human use, particularly solutions that go beyond personal protection to protect populations at scale, represent a national priority.

Spatial repellents (SRs), defined as “chemicals that when airborne prevent biting by blood seeking insects such as mosquitoes”, represent a potentially effective tool against vector-borne diseases (VBDs) (Arctec, 2020). There is growing interest in synthetic pyrethroids (SPs) – the only class of chemistry considered safe for prolonged human use – as SRs for personal protection against infective bites (Achee et al., 2012). Chemistries with known SR activity against insects include the volatile synthetic pyrethroids (SPs) metofluthrin and transfluthrin (Arctec, 2020). Transfluthrin (TF) is an effective SR in indoor settings against different species of mosquitoes, flies and sand flies, and confers protection from biting (Ogoma et al., 2017; Estrada et al., 2019; Mwanga et al., 2019; Britch et al., 2020a, b; Masalu et al., 2020). Randomized-controlled trials also suggest the potential for epidemiological impact on VBDs (Hill et al., 2014; Syafruddin et al., 2020). Several studies support the utility of SPs for personal protection against tick bites, delivered either *via* treated clothing or *via* clip-on device, and provided modest area protection (Bibbs and Xue, 2016a, Bibbs and Xue, 2016b; Eisen et al., 2017; Prose et al., 2018).

**Fig. 1:**
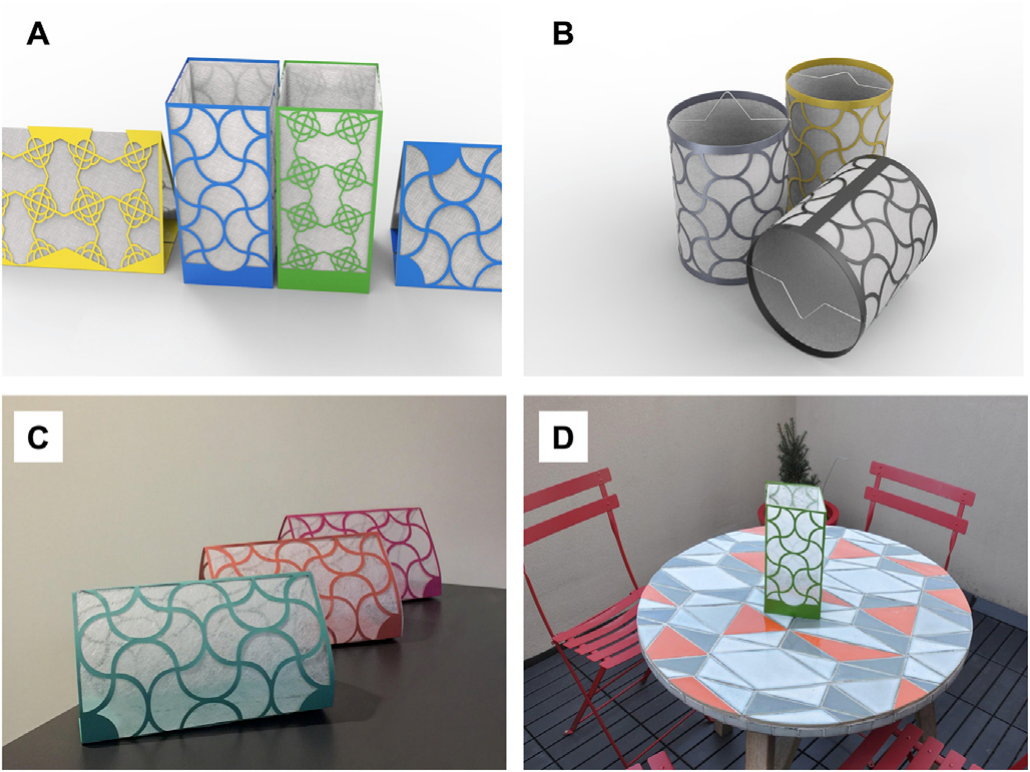
Image of the Personal Insect Repellent Kit (PIRK) showing surface (**A**) and lantern (**B**) configurations, and set-up (**C**-**D**).

At present there is no spatial or area tool on the market for killing ticks before they bite. The Personal Insect-Repellent Kit (PIRK) is an innovative new technology that could rapidly address this need and prevent infectious tick bites in a range of outdoor settings where civilians and the military have high risk of tick encounter. The PIRK is a small, lightweight, passive spatial device designed to protect against flying insects (Fig. 1). Developed by Widder Brothers Inc., the PIRK incorporates Bayonthrin (TF) *via* proprietary method. The PIRK has shown both knockdown (KD) and post-exposure mortality of three species of mosquitoes, sand flies and stable flies in an area of up to 30 m^3^ over periods up to three weeks. Initial outdoor studies have shown good efficacy against these same vectors*.* By repelling and killing a range of tick species, the PIRK could reduce (i) tick bites, (ii) tick populations, and (iii) the transmission of multiple tick-borne diseases, including diseases with the potential to affect deployed warfighters.

The present study was conducted to investigate the contact and spatial effects (if any) of the PIRK and AI, TF against three species of medical and veterinary importance: *I. scapularis*, *A. americanum* and *D. variabilis*. An initial assay series employing dose- and time-response was developed to assess the contact and spatial activity of the PIRK in an enclosed test system based on the parameters of percent KD and mortality. We explored the hypotheses that (i) tick response to the PIRK and TF following periods of continuous physical contact would be dependent on stage and species, and (ii) spatial activity would be evident after exposure of ticks at short range. An additional aim of this study was to observe the behavioral responses of tick species and stages to the PIRK on contact and at range.

## 2 Materials and methods

### 2.1 Ticks and chemicals

*Ixodesscapularis*, *A. americanum* and *D. variabilis* larvae, nymphs and adults were purchased from the Tick Rearing Facility, Department of Entomology and Plant Pathology, Oklahoma State University, USA. Transfluthrin and permethrin were purchased from Sigma-Aldrich (St. Louis, MO, USA). Treated and untreated PIRK was provided by study partner, Widder Brothers, Inc.

### 2.2 Larval immersion contact assay

The contact toxicity of TF to tick larvae was evaluated using a larval immersion assay, modified from White et al. (2004). Briefly, five serial dilutions of TF were prepared and 3 μl of each dose was added to the wells of a 96-well plate (Sigma-Aldrich) containing 97 μl ddH_2_O. Approximately 50 larvae were transferred to each well by sterile loop and immersed in test dose for 30 min. Larvae were then transferred to small (7 × 7 cm) surgical packets (Cancer Diagnostics Inc., Durham, NC, USA) again *via* pipet using wide bore tips and placed at room temperature inside a humidified chamber (relative humidity, RH, greater than 50%). Knockdown (KD), defined as “tick unable to walk/stand upright, but responding to touch and breath/heat stimulus” was assessed at 1 h post-exposure and mortality at 1, 24, 48, 72 and 96 h post-exposure. Minimum of *n* = 3 technical replicates were performed per dose and experiment. Permethrin (LC_90_ dose) and DMSO (3% final concentration) were used as positive and negative control, respectively. The LC_50_ at 24 h post-exposure was calculated using GraphPad Prism 8 software. The dose range varied by species and was selected following a series of pilot assays to ensure that doses spanned the expected LC_50_ value.

### 2.3 Adult/nymph contact assay

The efficacy of the PIRK to adults and nymphs was assessed *via* contact assay, modified from Eisen et al. (2017). Briefly, five ticks were introduced to a 10 cm in diameter Petri dish (Fisher Scientific, Waltham, MA, USA) containing a 10 cm diameter disk of the PIRK fabric. Ticks were placed in contact with the PIRK for 10, 20, 40, 80 and 120 s (*I. scapularis* adults and nymphs, and *A. americanum* nymphs) or 10, 40, 120, 180 and 240 s (*A. americanum* adults and *D. variabilis* adults and nymphs). The range of exposure periods was selected following a series of pilot assays to account for differences in the susceptibility of species. Following exposure, ticks were transferred to surgical packets and maintained at RT inside a humidified chamber for 96 h. KD defined as “tick unable to walk/stand upright, but responding to touch and breath/heat stimulus” was scored at 1 h and percent mortality at 1, 24, 48, 72 and 96 h post-contact. Ticks that exhibited the KD response at remaining timepoints in the assay were separately scored as “incapacitated”. Whatman paper #1 served as negative control. All assays were performed at a minimum of 70 °C and 50% RH and included a minimum of *n* = 3 technical replicates per exposure time for each stage and species, and *n* = 3 biological replicates. New PIRK fabric was used for each technical and biological replicate. GraphPad Prism 9.3.0 software and two-way ANOVA followed by Tukey’s multiple comparison test were used to compare the means of control and treatments for KD and mortality at each time-point post-exposure.

### 2.4 Modular spatial assay

**Fig. 2:**
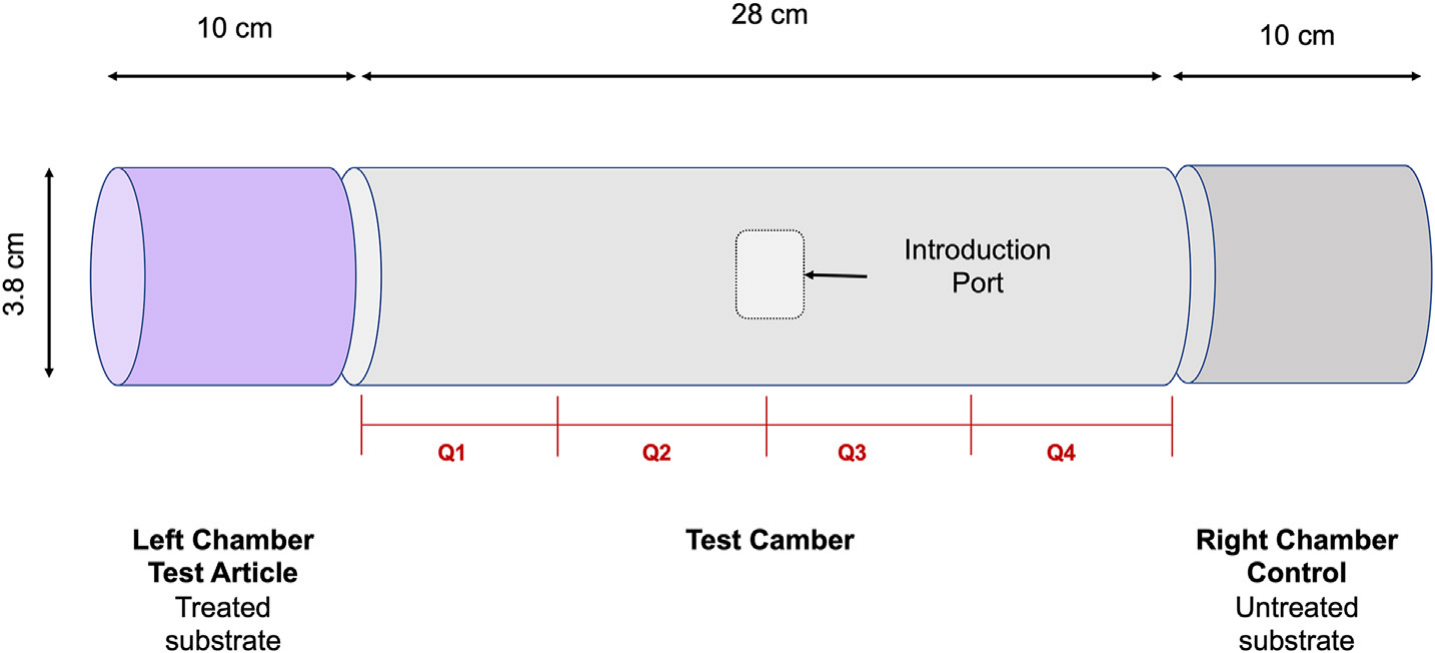
Schematic showing configuration of the spatial assay. The spatial activity of the Personal Insect Repellent Kit (PIRK) was investigated *via* a modular assay. The test apparatus consisted of three clear polycarbonate components: test chamber comprising quadrants Q1-Q4, separated from the left and right treatment/control chambers by mesh fabric. Treated and untreated (control) PIRK substrate was rolled inside the treatment/control chamber and permitted to acclimate under laboratory conditions for 0–2 h. Five adult female *I. scapularis* per technical replicate were placed inside the test chamber and physical location was recorded at 10-min intervals for up to 2 h, following which ticks were removed. Percent knockdown (KD) was recorded at 10-min intervals up to 1 h and mortality at 24 h, 48 h, 72 h and 96 h.

The spatial activity of the PIRK against *I. scapularis* adult females was investigated using a modular assay system modified from Grieco et al. (2005). The assay chamber comprised three components: a central cylinder (28 cm in length and 3.8 cm in diameter), flanked by two test drums (10 cm in length and 3.8 cm in diameter). All components were made of clear polycarbonate (Acuity Lighting Group Inc., Conyers, GA, USA) (Fig. 2). The treatment drums contained either untreated substrate (left and right drums) for the control group, or PIRK (left drum) and untreated PIRK substrate (right drum) for the treated group, separated from the test chamber by tulle fabric. The PIRK was permitted to acclimate for periods of 0 min, and 1 and 2 h, based on knowledge of AI release dynamics. Five ticks were introduced to the test chamber *via* a small, central port and position in the central cylinder was recorded every 10 min by image capture on a smart phone for up to 2 h. Tick behavior was also recorded *via* short videos on a smart phone. Ticks were then transferred to surgical packets and scored for KD every 10 min up to 1 h and for mortality at 1, 24, 48, 72 and 96 h. All assays were conducted at a minimum of 70 °C and 55% RH, and included a minimum of *n* = 3 technical replicates per assay and *n* = 3 biological replicates. All components of the test apparatus were replaced between treatment replicates and new PIRK fabric was used for each biological replicate. GraphPad Prism 9.3.0 software and two-way ANOVA followed by Tukey’s multiple comparison tests were used to compare the mean percentage of ticks located in each of the four quadrants at 10-min intervals during 2 h exposure.

## 3 Results

### 3.1 Larval immersion contact assay

**Fig. 3:**
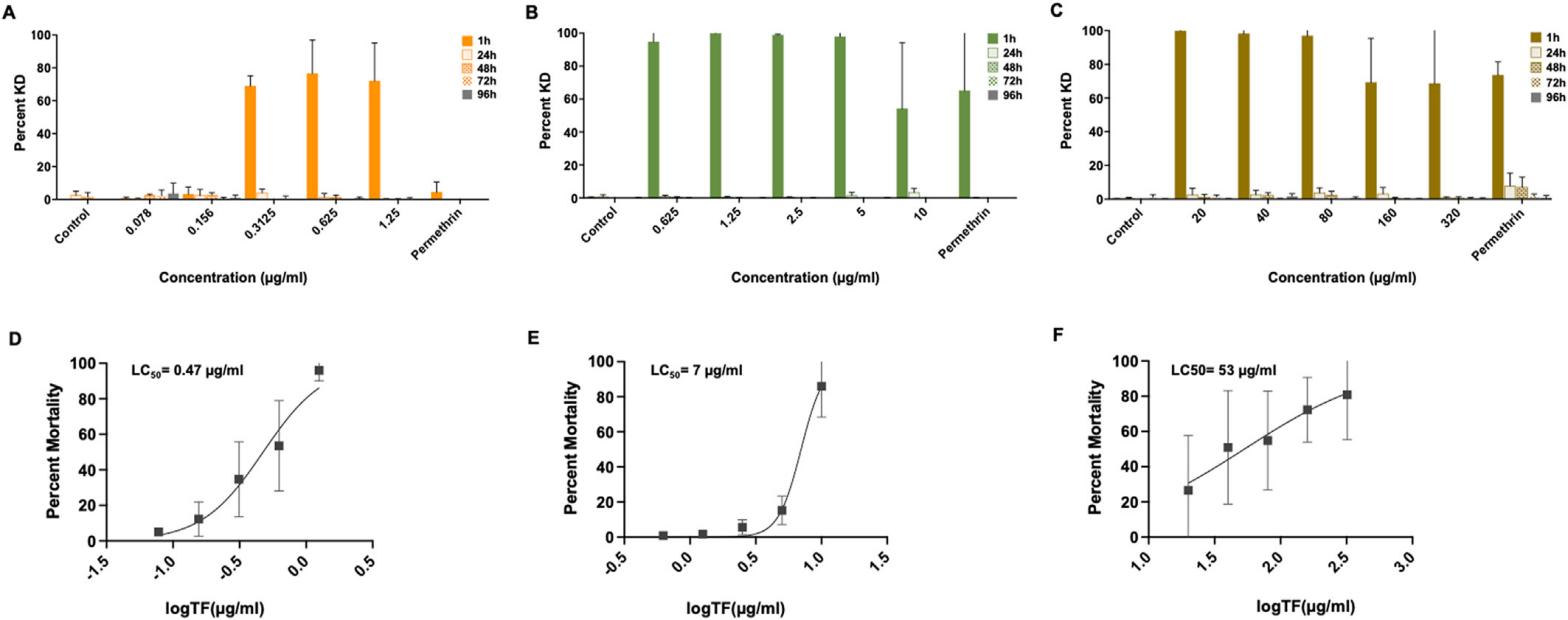
Efficacy of transfluthrin to *Ixodes scapularis*, *Amblyomma americanum* and *Dermacentor variabilis* larvae in a dose-response immersion assay. Graphs **A**-**C** show percent knockdown (KD) of larvae to at 1 h, 24 h, 48 h, 72 h and 96 h following a 30-min exposure to transfluthrin, relative to the positive control (permethrin, LC_90_ dose). Graphs **D**-**F** represent the dose-response of larvae at 24 h post-exposure. **A**, **D**, *Ixodes scapularis*; **B**, **E**, *Amblyomma americanum*; **C**, **F**, *Dermacentor variabilis.* Results represent *n* = 3 biological replicates.

*Ixodes scapularis*, *A. americanum* and *D. variabilis* larvae exhibited KD and mortality in the dose-response assay (Fig. 3; Supplementary Figure S1). Typical behaviors observed following exposure to TF included uncoordinated movement in the surgical packet (inability to crawl/orient) and movement of the legs following stimulus. TF was rapidly toxic to larvae of all species compared to the positive control, permethrin (LC_90_ dose). The toxicity of TF was dose-dependent with LC_50_ values of 0.47 μg/ml, 7 μg/ml and 53 μg/ml for *I. scapularis*, *A. americanum* and *D. variabilis*, respectively. The minimum TF dose required to achieve greater than 90% mortality of larvae at 24 h was 1.25 μg/ml, 10 μg/ml and 320 μg/ml for *I. scapularis*, *A. americanum* and *D. variabilis*, respectively. The minimum dose required to achieve greater than 90% KD at 1 h post-exposure was 0.625 μg/ml and 20 μg/ml for *A. americanum* and *D. variabilis*, respectively (Table 1). Greater than 90% KD of *I. scapularis* larvae was not observed under the conditions used for the immersion assay.

**Table 1:** Efficacy of transfluthrin to *Ixodes scapularis*, *Amblyomma americanum* and *Dermacentor variabilis* larvae as assessed in the larval dose-response contact assay

Efficacy	*I. scapularis*	*A. americanum*	*D. variabilis*
LC_50_	0.47 μg/ml	7 μg/ml	53 μg/ml
Minimum dose to achieve KD ≥ 90% at 1 h post-exposure^a^	–	0.625 μg/ml	20 μg/ml
Minimum dose to achieve mortality ≥ 90% at 24 h post-exposure^a^	1.25 μg/ml	10 μg/ml^b^	320 μg/ml^c^

*Notes*: Results show the lethal concentration (LC_50_, 24 h) and minimum dose TF required to achieve greater than 90% knockdown (KD) and mortality at 1 h and 24 h, respectively for each species. Data represent minimum three biological replicates (*n* = 3).

a Based on mean mortality/KD.

b 48 hours.

c 96 hours.

### 3.2 Adult/nymph contact assay

**Fig. 4:**
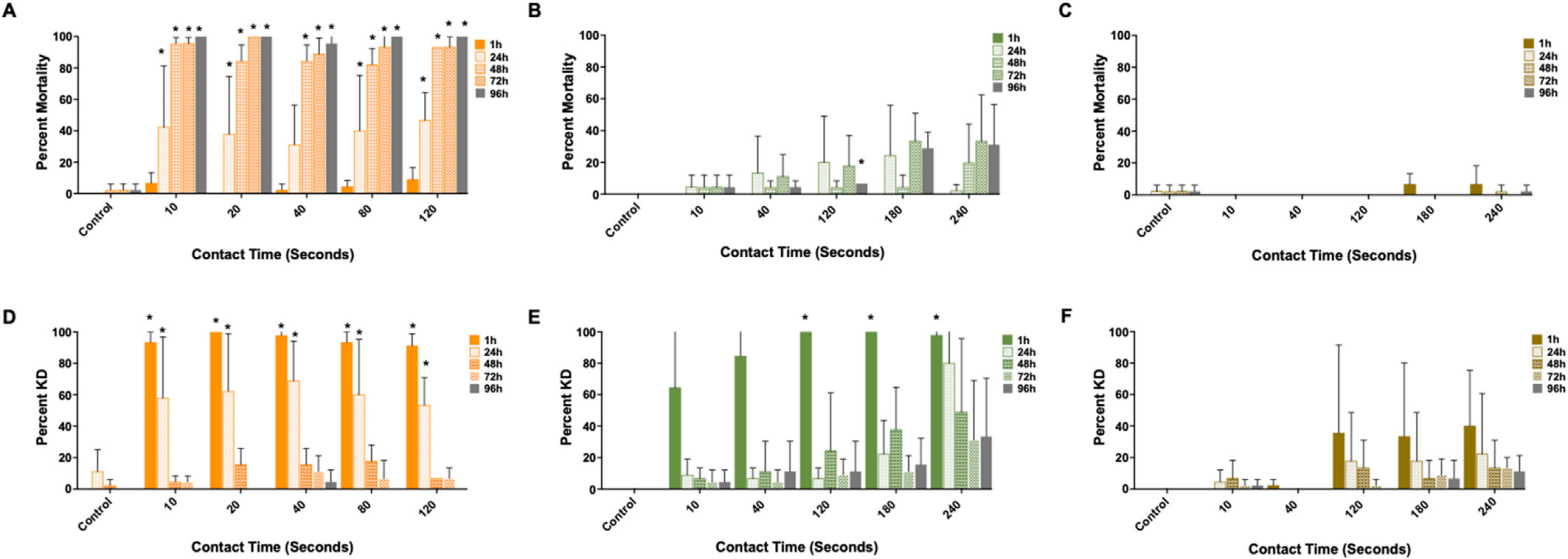
Efficacy of the Personal Insect Repellent Kit (PIRK) to *Ixodes scapularis*, *Amblyomma americanum* and *Dermacentor variabilis* adults in forced-exposure contact assay. Graphs show percent mortality (**A-C**) and knock-down (KD) (**D-F**) at 1 h, 24 h, 48 h, 72 h and 96 h following contact with the PIRK substrate for 10–240 s in an enclosed test chamber. **A**, **D**, *Ixodes scapularis*; **B**, **E**, *Amblyomma americanum*; **C**, **F**, *Dermacentor variabilis.* Results represent *n* = 3 biological replicates. Statistical significance (*P* < 0.05) is indicated by a symbol on top of the bar graph: asterisk indicates statistical significance between PIRK-exposed and control group at the indicated post-exposure time.

**Fig. 5:**
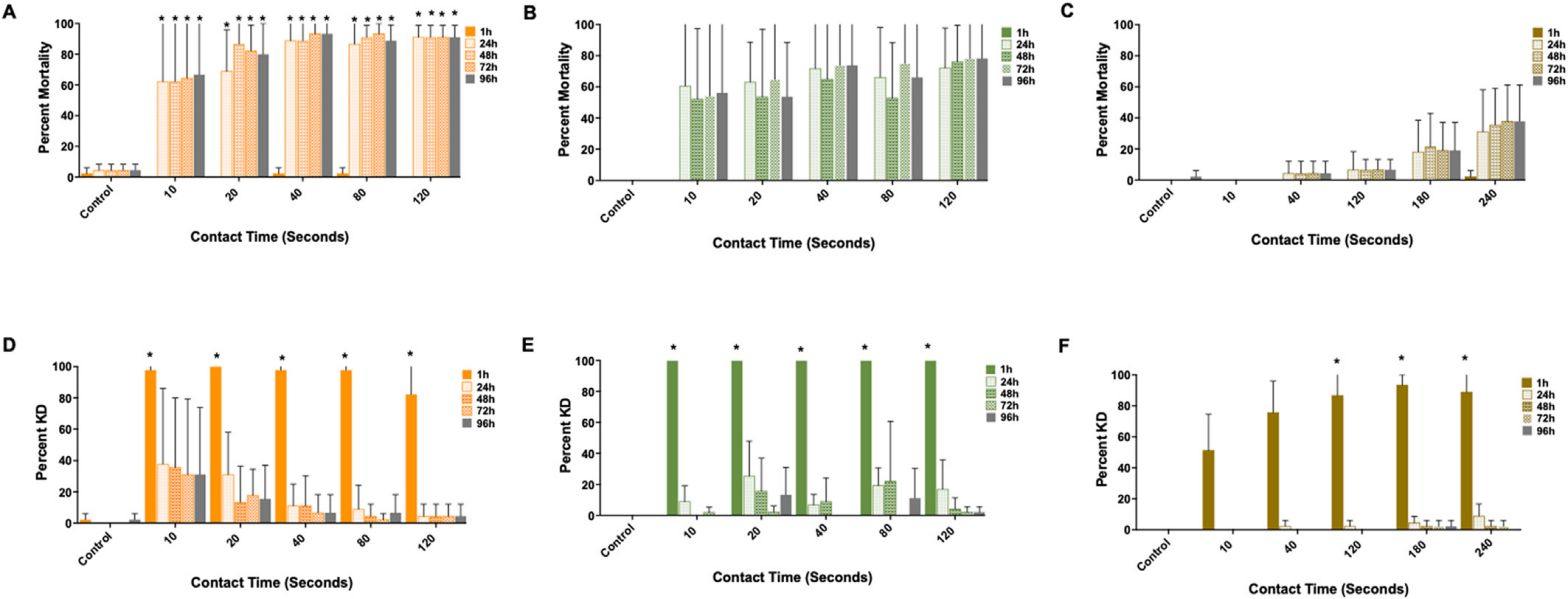
Efficacy of the Personal Insect Repellent Kit (PIRK) to *Ixodes scapularis*, *Amblyomma americanum* and *Dermacentor variabilis* nymphs in forced-exposure contact assay. Graphs show percent mortality (**A-C**) and knock-down (KD) (**D-F**) at 1 h, 24 h, 48 h, 72 h and 96 h following direct contact with the PIRK substrate for 10–120 s in an closed test chamber. **A**, **D**, *Ixodes scapularis*; **B**, **E**, *Amblyomma americanum*; **C**, **F**, *Dermacentor variabilis.* Results represent *n* = 3 biological replicates. Statistical significance (*P* < 0.05) is indicated by a symbol on top of the bar graph: asterisk indicates statistical significance between PIRK-exposed and control group at the indicated post-exposure time.

*Ixodes scapularis*, *A. americanum* and *D. variabilis* adults and nymphs exhibited KD and mortality following contact with the PIRK in an enclosed assay system (Fig. 4, Fig. 5; Table 2). Behavioral responses consistent with irritancy and avoidance were observed in all species and included the “hot foot effect”, whereby ticks attempted to move off the substrate by crawling on the side or lid of the Petri dish, or beneath the fabric. This effect was observed only when the substrate was in contact with the tarsi and not the dorsum, i.e. when ticks crawled beneath the fabric. Onset of the hot foot effect was slowest in *D. variabilis* adults which were frequently observed clustered together, including on the sides of the Petri dish, or were inactive with their legs retracted beneath the ventral scutum. Several phenotypes persisted following removal of the tick from contact with the PIRK: inability to orient/stand-upright, uncoordinated/side-ways movement, and infrequently, hypostome/pedipalp presentation. These phenotypes were observed in *I. scapularis* and *A. americanum* adults and nymphs, *D. variabilis* nymphs and in a few instances, *D. variabilis* adults. These phenotypes were not observed in adults and nymphs of the negative control, which exhibited typical questing behavior (forward movement and presentation of the forelegs). Questing behaviors were pronounced in *A. americanum* and observed infrequently in *D. variabilis*. Greater than 90% KD was achieved after a minimum exposure of 10 and 120 s in *I. scapularis* and *A. americanum* adults, respectively, and 10 s in the nymphs of both species. *Dermacentor* *variabilis* nymphs exhibited greater than 90% KD after a minimum contact-exposure of 180 s. This threshold was not observed in the case of *D. variabilis* adults at any contact-exposure time employed for this assay. The minimum exposure time required to achieve greater than 90% mortality was 10 and 40 s in *I. scapularis* nymphs and adults, respectively.

**Table 2:** Efficacy of the PIRK to *Ixodes scapularis*, *Amblyomma americanum* and *Dermacentor variabilis* adults and nymphs as assessed in the contact assay

Species/Stage	*I. scapularis*	*A. americanum*	*D. variabilis*
Minimum exposure time to achieve KD ≥ 90%, 1 h post-exposure			
Adults	10 s	120 s	ND
Nymphs	10 s	10 s	180 s
Minimum exposure time to achieve mortality ≥ 90%, 24 h post-exposure			
Adults	10 s^a^	ND	ND
Nymphs	40 s	ND	ND

*Notes*: Results show the minimum exposure time required to achieve greater than 90% knockdown (KD) at 1 h and 90% mortality at 24 h post-exposure, respectively for each species. Data represent minimum three biological replicates (*n* = 3).

*Abbreviation*: ND, not determined.

a 48 hours.

### 3.3 Modular spatial assay

**Fig. 6:**
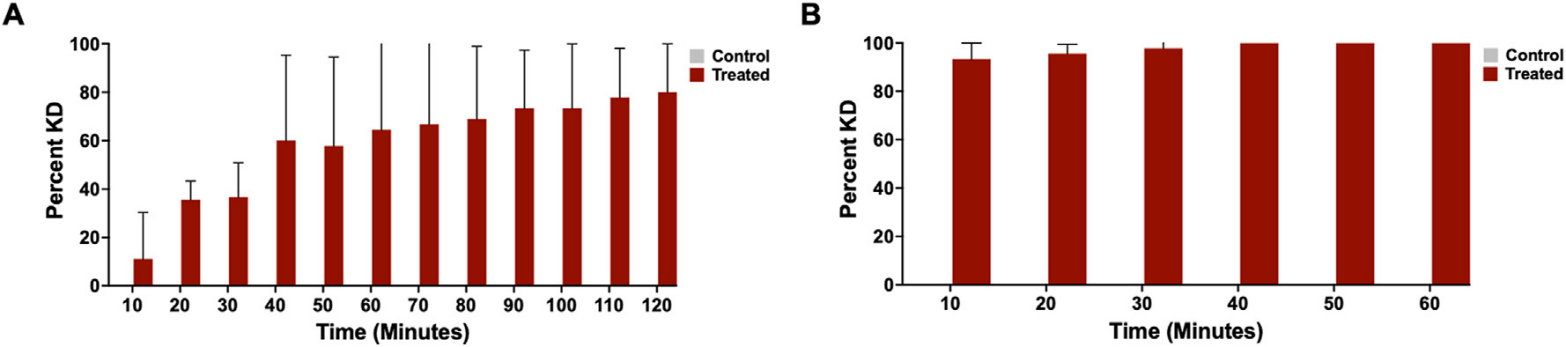
Spatial efficacy of the Personal Insect Repellent Kit (PIRK) to female *Ixodes scapularis* adults. Graphs show percent knockdown (KD) of ticks at 10-min intervals over 120-min exposure to the PIRK (**A**) and at 10–60 min following removal form the test chamber (**B**). Results represent *n* = 3 biological replicates. Error bars at 100% not shown.

**Fig. 7:**
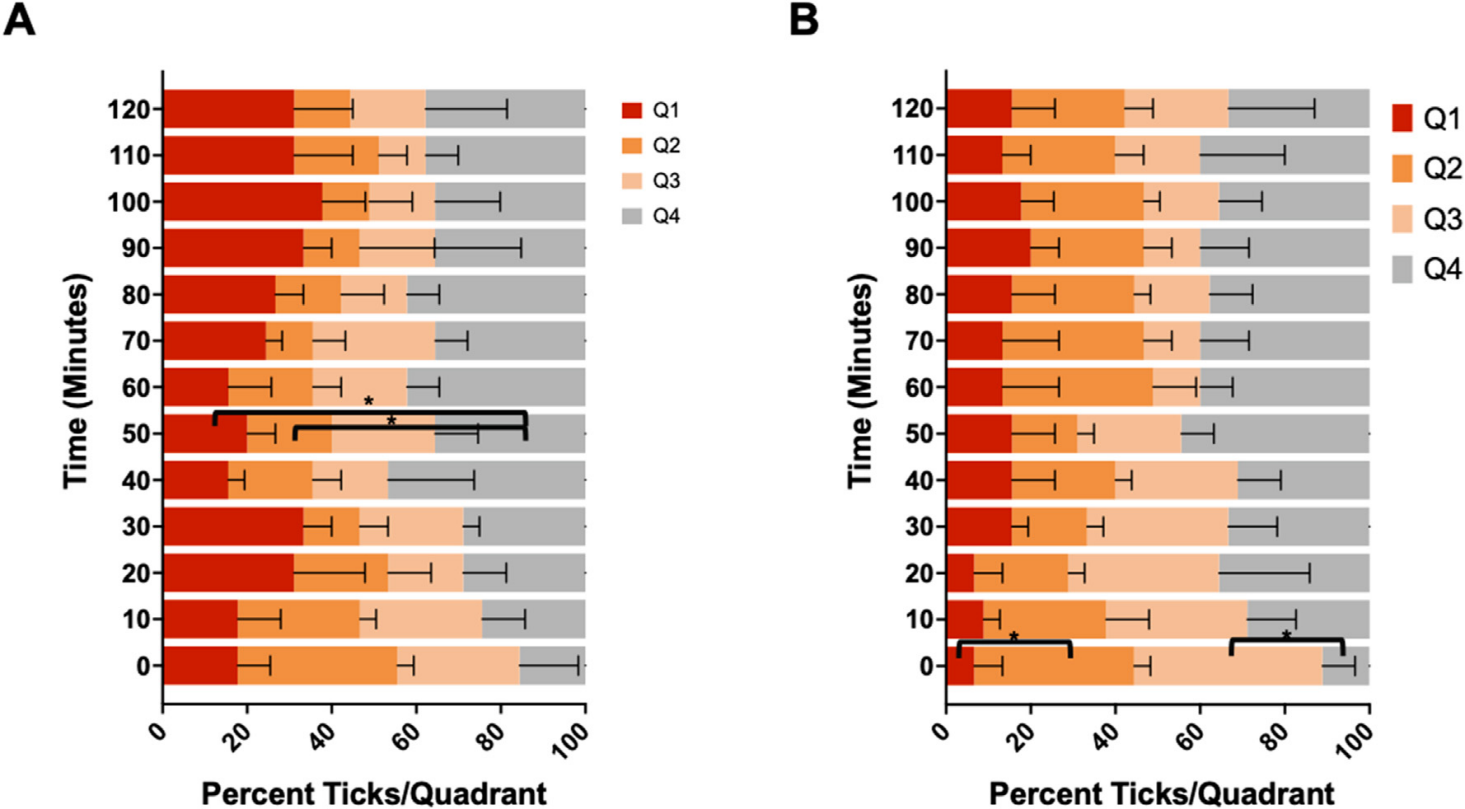
Spatial efficacy of the Personal Insect Repellent Kit (PIRK) to female *Ixodes scapularis* adults. Graphs show the percentage of ticks located in each of four quadrants of the modular test apparatus at 10-min intervals over the course of 120-min exposure to the control (untreated substrate in left and right test chambers) (**A**) and the test article (PIRK *versus* untreated substrate in left and right chambers) (**B**). Results represent *n* = 3 biological replicates. Statistical significance (*P* < 0.05) is indicated by an asterisk.

**Fig. 8:**
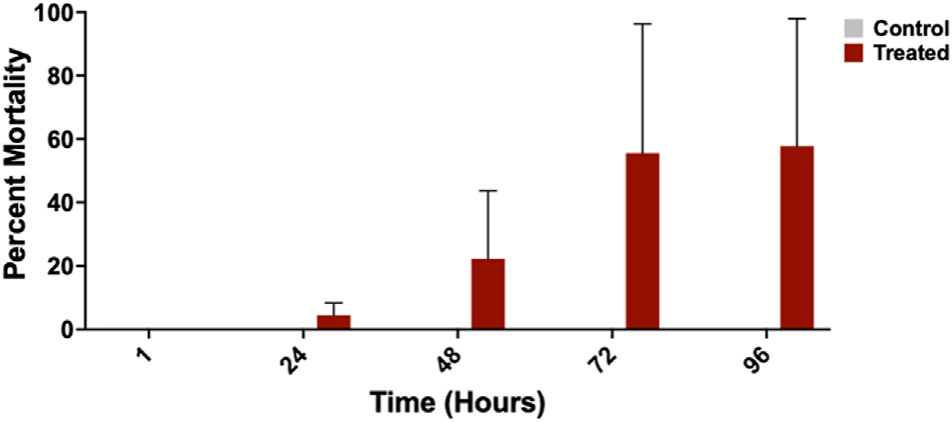
Spatial efficacy of the Personal Insect Repellent Kit (PIRK) to female *Ixodes scapularis* adults. Graph shows the percent mortality of ticks at 1 h, 24 h, 48 h, 72 h and 96 h that were exposed to the PIRK for a total of 120 min. Results represent *n* = 3 biological replicates.

An enclosed, modular assay system was used to assess the spatial activity of the PIRK against *I. scapularis* adult females (Fig. 2). The assay has been used to test response of mosquitoes to AI in vapor phase, following passive release from the test substrate and was adapted here for tests with ticks. Pilot assays were performed to determine the optimal exposure time required to observe spatial effects (0, 60 and 120 min acclimation of the PIRK before introduction of ticks; data not shown) and an acclimation period of 120 min was selected for experiments. Pilot assays also investigated a variety of exposure intervals (1–10 min) and total exposure times (10 min, 30 min, 1 h and 2 h) (data not shown). Scoring intervals of 10 min and a total exposure time of 2 h was selected. The spatial assay revealed irritation and KD of adult *I. scapularis* females (Fig. 6A) in chamber. KD was first detected in chamber at 10 min, with the majority of ticks unable to stand upright at a 120 min exposure. Some ticks were observed pivoting in multiple directions with forward presentation and rapid movement of the forelegs. These effects correlated with the length of exposure in chamber. Evidence of attraction/repellency (movement towards or away from the test drum) was not observed, and ticks in both control and treated groups were recorded in all quadrants of the test arena (Fig. 7A-B). Some ticks were observed at the end of the test arena (treated or control end) crawling on the tulle fabric. KD was observed in more than 80% of ticks in chamber at a 120-min exposure (Fig. 6A). Following removal from the chamber, more than 90% KD of ticks was observed at 10 min post-exposure with 100% KD at 1 h (Fig. 6B). High variability in mortality post-exposure was observed within and between biological replicates and on average, greater than 50% mortality of ticks was observed at 72 h post-exposure (Fig. 8).

## 4 Discussion

At present, civilians and military personnel lack non-wearable spatial devices to repel and/or kill ticks and prevent infectious bites. The development of new SR technologies for tick control requires the development of efficacy protocols for regulatory approval. National regulatory approval of insecticidal SRs for the consumer market has utilized parameters of KD and mortality (Arctec, 2020) and the WHO has developed guidelines for assessment of SRs for vector control (WHO, 2013). The terrestrial habitat of hard ticks, including host questing and location from the ground or low-level vegetation, are factors that must be considered in the design of efficacy studies to evaluate SRs as bite-prevention technologies. Here, we present the first study to investigate the efficacy of the PIRK, a passive spatial device that has shown efficacy against flying pests, for potential to prevent human-tick encounters and protect from tick-bites. Studies focused on three species of ixodid (hard) ticks of importance to military and civilian health: the black legged tick, *I. scapularis* (vector of Lyme disease, anaplasmosis, babesioisis and Powassan virus), the lone star tick, *A. americanum* (vector of ehrlichiosis, tularemia, heartland and Bourbon viruses) and the American dog tick, *D. variabilis* (vector of Rocky Mountain spotted fever and tularemia).

The larval immersion assay of White et al. (2004) was used to establish the contact efficacy of TF, the AI used in the PIRK, to tick species. Larval dose-response data provided a basis to interpret contact efficacy studies employing the PIRK. Based on LC_50_ value, the efficacy of TF to larvae was as follows (highest to lowest): *I. scapularis* > *A. americanum* > *D. variabilis*. Results are consistent with the study of White et al. (2004) who reported an evaluation of multiple acaricides against species of hard ticks and found that *D. variabilis* in particular, was less susceptible to all acaricides tested except in the case of the organophosphate, chlorpyrifos. The variation observed in potency of TF between tick species likely reflects biomass and physiological differences.

Nymphs and adults of *I. scapularis* and *A. americanum,* and nymphs of *D. variabilis* exhibited KD at 1 h following short (10–180 s) periods of forced exposure to the PIRK in the contact assay. Ticks exhibited irritation and avoidance behaviors, as reported in other studies of tick response to SPs (Eisen et al., 2017; Prose et al., 2018), and were incapable of orientation and lateral movement, suggesting potential for PIRK embodiments to disrupt host location. Mortality was observed at 24 h in *I. scapularis* nymphs and adults exposed to the PIRK for 10–40 s. Mortality of *A. americanum* was not observed under assay conditions, despite 100% KD of this species at exposure times exceeding 120 min, suggesting that longer periods of exposure would be required to achieve lethal dose. KD of *D. variabilis* adults was not achieved in the assay, including at contact periods up to 4 min. These results agree with larval immersion data and suggest that *D. variabilis* is the least susceptible of the three species to TF and the PIRK. Longer exposure times would be required to establish the potential of the PIRK against adult *D. variabilis* but may be unrealistic for certain field situations. These data support greater potency of the PIRK to immature tick stages and the prostriate tick, *I. scapularis*, a phenomenon also noted by Prose et al. (2018) in studies of ticks exposed to SP-treated fabric.

**Fig. 9:**
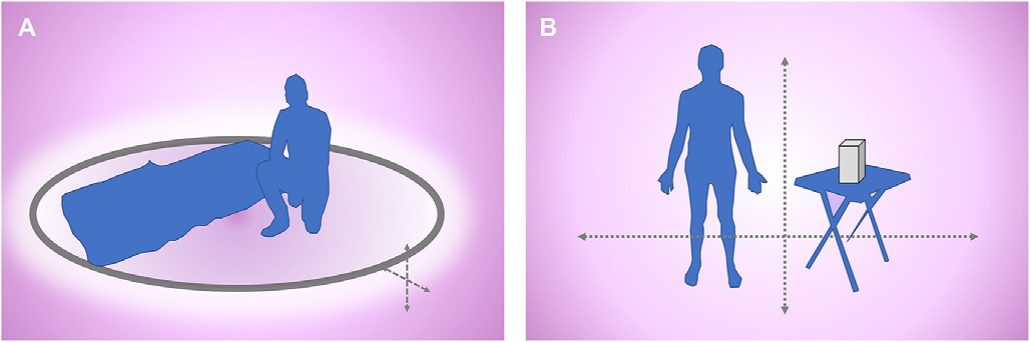
Concept of non-wearable spatial tick-bite prevention technologies. The terrestrial activity of ticks necessitates unique approaches to the design of non-wearable bite-prevention devices for limited outdoor use. Schematic showing concepts for: **A** Static continuous perimeter device such as for temporary ground use; **B** Table-top or lantern-style device such as for camping or in garrison. Efficacy studies must determine the size of the 3-dimensional area protected and the minimum period of protection, including under conditions of ambient wind, temperature, sunlight, relative humidity and barometric pressure.

Data from the forced contact studies suggest potential of the PIRK to provide a physical barrier to questing ticks in ground use scenarios (Fig. 9A). Further, in addition to control of ticks that contact the substrate for short periods, continued deployment of the PIRK in limited outdoor settings (such as in camps or garrison) could suppress local tick populations. Differences were noted in the behavioral responses of tick species on exposure to the PIRK that could have relevance for the design of PIRK prototypes and other tick-bite prevention strategies. The propensity of ticks to crawl beneath and limit tarsal contact with the PIRK, the clustering behavior of *D. variabilis* and the aggressive questing behavior of *A. americanum*, including speed/distance traveled relative to other species, are factors which could affect the delivery of an effective dose. Although not investigated here, development of the PIRK as a physical barrier device will also require determination of the minimum dimensions (H × W × D) that prevent human-tick encounters, especially under field conditions. Designs may need to consider contact irritancy and hyper-excitation, factors which could contribute to dislodgement of the tick from the TF-treated substrate (Prose et al., 2018).

An enclosed modular assay system was developed to rapidly evaluate spatial efficacy of the PIRK under forced exposure and controlled conditions. The assay was modified from that of Grieco et al. (2005) used to assess the spatial activity index (SAI) of volatile AIs to mosquitoes. Initial assessment focused on adult female *I. scapularis* as this species exhibited greatest sensitivity to TF and the PIRK in contact assays and is responsible for transmission of Lyme disease, the most common VBD in the USA and Europe. KD was observed starting at 10 min in ticks exposed at short range (5–28 cm) to the PIRK, presumably in response to volatile TF molecules released into the test chamber, and high levels of KD (greater than 90%) persisted for up to 1 h. Post-exposure mortality was also observed in ticks following a 120-min exposure in the test chamber. The observed KD and mortality support the efficacy of the PIRK as an insecticidal SR. We hypothesize that the KD observed in chamber, and post-exposure KD and mortality, resulted from exposure to TF in vapor phase. However, we recognize that the residual properties of synthetic pyrethroids such as TF, combined with the terrestrial lifestyle of ticks (i.e. tarsi in contact with a treated substrate) could contribute to dermal exposure, and these factors should be considered when evaluating spatial activity. Questions remain regarding the contribution of dermal *versus* inhalation exposure (i.e. *via* the spiracles) to the observed KD and mortality, as well as how detoxification processes associated with these routes might impact efficacy. Of note, ticks in fasting stage (as employed in our assays) are known to have a discontinuous gas exchange, regulated by the intermittent opening of the spiracles, and characterized by brief bursts of CO_2_ with open spiracles followed by long periods (about 1 h or more depending on the species) with low CO_2_ emission where the spiracles are likely closed (Fielden et al., 1999). Therefore, the potential for amplification of AI intake through this route is not clear in the case of ticks. Ongoing studies are focused on evaluation of PIRK insecticidal SR activity against additional life stages, sexes and species of ixodid ticks.

Tick movement towards or away from the test article was not observed in the spatial assay, although at a 20-min exposure some ticks were noted on the mesh fabric separating the test arena from the treatment and control chambers. Similar results were obtained without acclimation of the PIRK in pilot studies (data not shown). The efficacy of pyrethroids as both repellents and toxicants, depending on dose, is well established (Bibbs and Xue, 2016a, Bibbs and Xue, 2016b; Marino-Gomez et al., 2021). The apparent lack of repellency observed in the modular assay may reflect saturation of the test chamber with TF, but given the results of pilot studies, failure to establish a TF chemical gradient is considered an unlikely explanation. Both olfaction and taste are thought to mediate repellency in blood-feeding vectors (Dennis et al., 2019). Long- and short-range detection and acquisition of TF and other SPs presumably occurs mostly through the tarsi in ticks, and mechanisms of AI acquisition and repellency represent important areas for further investigation. Ongoing spatial activity studies are focused on determination of the minimum exposure time and distance in test chamber required to achieve 90% KD 1 h post-removal, as well as the response of ticks over longer test distances, under conditions of unidirectional air flow and in “open” small cage and semi-field systems. These experiments are expected to permit comparative assessments of PIRK efficacy with studies of commercial devices (see Bibbs and Xue, 2016a, Bibbs and Xue, 2016b for example) and possibly, the determination of a deterrent chemical dose.

By definition, SRs are airborne chemicals that disrupt host biting. Studies are ongoing to establish the area of spatial protection provided by the PIRK and the minimum time and distance required for greater than 90% KD under controlled conditions. In future work, it will be important to evaluate the capabilities of PIRK prototypes under semi-field conditions where variations in ambient temperature, relative humidity, sunlight, air pressure and relative wind speed will impact TF release and dissemination. The assays described herein evaluated the PIRK against ticks in the absence of a human stimulus in order to establish a baseline for contact and spatial efficacy. An assessment of PIRK efficacy in the presence of host cues such as CO_2_, and possibly employing modified WHO SR assays and involving human subjects, will be important to demonstrate bite prevention capabilities (WHO, 2013).

Substantial biological variation between ticks employed for technical and biological replicates was observed in all assays and could result from factors such as tick age, rearing conditions and genetics. Such variables are difficult to control given the complexities of tick culture and the lack of highly in-bred tick cultures, but results argue the importance of robust experimental design and replication. Future studies should consider evaluation of efficacy using field-collected material in order to clarify differences that have been noted between the response of laboratory reared *versus* field collected ticks (Eisen et al., 2017; Prose et al., 2018).

The present study adds to understanding of SRs for control of ticks and TBDs. Our findings are comparable with those of Prose et al. (2018) who noted disrupted activity of *I. scapularis*, *A. americanum* and *D. variabilis* at 1 h following short (1–2 min) forced exposures to permethrin-treated clothing. The findings are also in line with the study of Bibbs & Xue (2016a) who observed delayed mortality of *A. americanum* nymphs at 24 h following 15–60 min forced exposures of ticks to the volatile SPs, allethrin and metofluthrin released from the ThermaCell and OFF! Clip-on devices, and greatest mortality of ticks exposed at short ranges.

## 5 Conclusions

We describe the first study to evaluate the efficacy of the PIRK against three ixodid tick species, representative of genera that pose high risk to civilians, military in garrison and the deployed war fighter. The data support efficacy (KD and post-exposure mortality) following short (10–120 s) periods of physical contact with the PIRK, and suggest potential to provide immediate barrier protection from questing ticks (Fig. 9A). Contact efficacy was species- and stage-specific, and was greatest to the nymphs and adults of *I. scapularis*, the vector of Lyme disease and other TBDs in the USA. Results also suggest spatial activity of the PIRK and potential for delivery of an insecticidal SR (Fig. 9B). KD of adult *I. scapularis* was observed in a test chamber at short (5–28 cm) range from the PIRK starting at 10 min, and greater than 90% KD occurred as early as 10 min in ticks removed from chamber following an exposure period of 120 min. High levels of mortality at 24 h in ticks exposed *via* contact or at range suggest that the PIRK could also contribute to tick control. Results from this initial experimental series will have bearing on the design of devices for both military and civilian applications.

## Funding

This work was supported by Department of Defense, Deployed War Fighter Program (DWFP) program funds, W911QY1910009 to CAH and LW.

## Ethical approval

This study does not require ethical approval.

## CRediT author statement

Maria V. Murgia: methodology, formal analysis, investigation, writing-original draft, review and editing, visualization. Jasleen Kaur: investigation. Laurie Widder: conceptualization, resources, writing-review and editing. Catherine A. Hill: conceptualization, methodology, validation, writing-original draft, review and editing, visualization, supervision, funding acquisition.

## Declaration of competing interests

The authors declare that they have no known competing financial interests or personal relationships that could have appeared to influence the work reported in this paper.

## Appendix A Supplementary data

Multimedia Comp 1 Multimedia Comp 1
