# Supplementary material for: Efficacy of the transfluthrin-based personal insect repellent kit (PIRK) against the ixodid ticks Ixodes scapularis, Amblyomma americanum and Dermacentor variabilis
Source: Curr Res Parasitol Vector Borne Dis. 2021 Dec 20;2:100070. doi: 10.1016/j.crpvbd.2021.100070 (PMC9795340; doi:10.1016/j.crpvbd.2021.100070)
Supplement: Multimedia Comp 1 [file mmc1.docx]

**
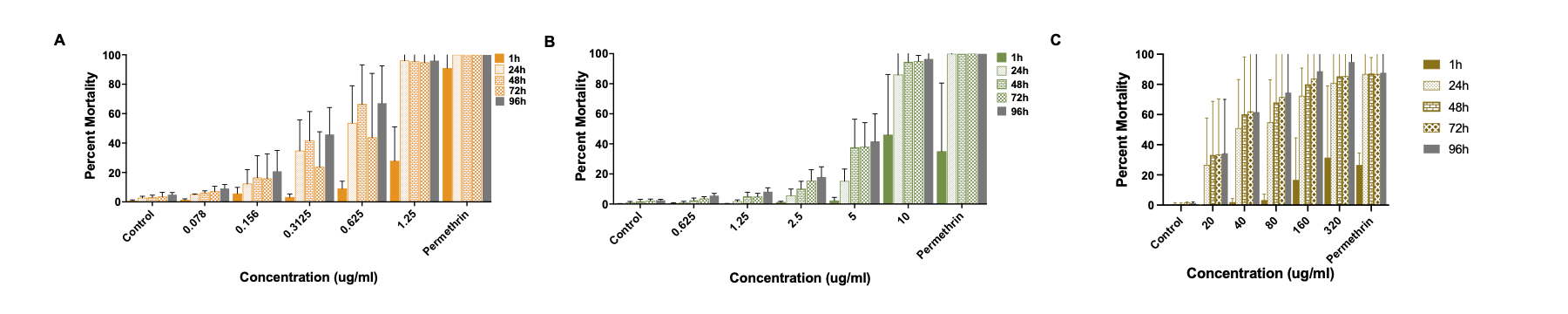
**

**Supplemental Figure S1. Efficacy of transfluthrin to *Ixodes scapularis*, *Amblyomma americanum* and *Dermacentor variabilis* larvae in a dose-response immersion assay.** Graphs show percent mortality of *Ixodes scapularis* (**A**), *Amblyomma americanum* (**B**) and *Dermacentor variabili* (**C**) larvae to transfluthrin at 1, 24, 48, 72 and 96-hours following a 30 minute exposure to transfluthrin, relative to the positive control (permethrin, LC_90_ dose). Results represent n=3 biological replicate.
